# Domain-based small molecule binding site annotation

**DOI:** 10.1186/1471-2105-7-152

**Published:** 2006-03-17

**Authors:** Kevin A Snyder, Howard J Feldman, Michel Dumontier, John J Salama, Christopher WV Hogue

**Affiliations:** 1 The Blueprint Initiative, 200 Elm St., Suite 101, Toronto ON, M5T 1K4, Canada; 2 Department of Biology, Carleton University, 1125 Colonel By Drive, Ottawa ON, K1S 5B6, Canada; 3 Samuel Lunenfeld Research Institute, Room 1060, Mount Sinai Hospital, 600 University Ave., Toronto, Ontario, M5G 1X5, Canada

## Abstract

**Background:**

Accurate small molecule binding site information for a protein can facilitate studies in drug docking, drug discovery and function prediction, but small molecule binding site protein sequence annotation is sparse. The Small Molecule Interaction Database (SMID), a database of protein domain-small molecule interactions, was created using structural data from the Protein Data Bank (PDB). More importantly it provides a means to predict small molecule binding sites on proteins with a known or unknown structure and unlike prior approaches, removes large numbers of false positive hits arising from transitive alignment errors, non-biologically significant small molecules and crystallographic conditions that overpredict ion binding sites.

**Description:**

Using a set of co-crystallized protein-small molecule structures as a starting point, SMID interactions were generated by identifying protein domains that bind to small molecules, using NCBI's Reverse Position Specific BLAST (RPS-BLAST) algorithm. SMID records are available for viewing at . The SMID-BLAST tool provides accurate transitive annotation of small-molecule binding sites for proteins not found in the PDB. Given a protein sequence, SMID-BLAST identifies domains using RPS-BLAST and then lists potential small molecule ligands based on SMID records, as well as their aligned binding sites. A heuristic ligand score is calculated based on E-value, ligand residue identity and domain entropy to assign a level of confidence to hits found. SMID-BLAST predictions were validated against a set of 793 experimental small molecule interactions from the PDB, of which 472 (60%) of predicted interactions identically matched the experimental small molecule and of these, 344 had greater than 80% of the binding site residues correctly identified. Further, we estimate that 45% of predictions which were not observed in the PDB validation set may be true positives.

**Conclusion:**

By focusing on protein domain-small molecule interactions, SMID is able to cluster similar interactions and detect subtle binding patterns that would not otherwise be obvious. Using SMID-BLAST, small molecule targets can be predicted for any protein sequence, with the only limitation being that the small molecule must exist in the PDB. Validation results and specific examples within illustrate that SMID-BLAST has a high degree of accuracy in terms of predicting both the small molecule ligand and binding site residue positions for a query protein.

## Background

Finding a protein sequence with small molecule binding site annotation can be a challenge as these are not consistently well annotated in existing sequence databases, regardless as to the degree of sequence similarity to proteins of known structure with well characterized small molecule binding sites. When annotation does exist, it may come from one of three places: directly from experiment, through mapping to a protein family known to bind certain molecules, or through homology to a crystal structure demonstrating small molecule binding.

Experimentally, small molecule ligands for proteins are often identified using high-throughput screening methods with chemical libraries. Such libraries may be developed in either solid or solution phase and can consist of natural compounds and their derivatives or synthetic molecules [1]. Chemical libraries are often generated using combinatorial chemistry, whereby both functional groups and molecular skeletons of precursor compounds are sequentially altered [2,3]. High-throughput screening has proven to be useful, especially in the areas of drug discovery [4,5] and food research [6]. The importance of high throughput screening is underscored by the fact that roughly 14% of the total research and development expenditures of the pharmaceutical industry is devoted to it [7]. Much of this effort is going towards increasing the number of molecules that can be screened at a time. However, the quality of a high throughput screen is likely to be more important than the quantity of ligands, as the number of false-positives seems proportional to the size of the library. The development of more directed screens, smaller-scale assays that can be run less frequently, is desired for a number of reasons, not the least of which is that the cost of a single high throughput screen is approximately US $75,000. [7] To achieve this end, *in silico *screening has been developed to search a large virtual library of compounds for a limited number of candidate molecules that can be tested further using more traditional means [8].

In addition to knowing binding partners, having accurate binding site information can greatly reduce the complexity of computational drug docking, for example. With a known binding site where a putative drug will bind, the algorithm need only explore the conformational space of the ligand in the vicinity of the binding site, and not over the entire surface of the protein. Protein-small molecule interaction databases are essential in the development of more advanced heuristic methods. These databases contain information about binding sites [9-11], electrostatics at the interface [12] or binding pocket information [13]. The majority of these are generated from data in the Protein Data Bank (PDB) [14] database of known protein crystal structures. For many of these interactions, binding affinity is often available in separate publications, and it is possible to construct training and test sets for computational drug docking algorithms.

While a wealth of information on small molecule binding is available in the PDB and given extensive work on protein family databases [15-17], the tools do not exist to make use of this information and map it to annotation of protein sequences in a consistent and efficient fashion. UniProt [18] has begun to add small molecule binding site annotation based on similarity to PDB sequences bound to small molecules. Only sites of very high confidence are added however, and annotation seems to be incomplete. For example, a search for human immunodeficiency virus 1 (HIV-1) integrase turns up a record (Q77Y09_9HIV1) including keywords 'zinc' and 'zinc-finger', and a link to the PDB file 1WJB, which is an NMR structure of the N-terminal domain of this protein with two bound zinc ions. However, no zinc binding sites are indicated in the record itself. Relibase [19] is able to provide small molecule binding site predictions for given query sequences based on interactions observed from PDB. However it makes no attempt to filter out biologically irrelevant ones (such as those with solvent), and often gives very lengthy, unranked output. PRECISE [20] clusters together similar PDB sequences for enzymatic proteins and maps the ligand binding sites to all members of each cluster, but does not allow ligand prediction for molecules not in PDB. Sequences Annotated by Structure (SAS) [21] uses FASTA [22] to align query sequences to known structures and add small molecule binding annotation, however it is unable to detect more remote homologs that can be found with more recent and sensitive methods such as PSI-BLAST [23]. The ability to detect distant homology allows more small molecule annotation to be added, or allows it to be added when methods like FASTA or BLAST against PDB return no significant hits. While all these methods rank the hits found to PDB, no attempt is made to give confidence values for the individual small molecule predictions, often resulting in far too many hits for a human to sit down and sift through.

One other important flaw these other methods share is the potential for transitive annotation error, as any molecule found in a PDB file that 'hits' the query is output, even if it binds in a region outside of the alignment (SAS does show which regions of the hits align to the query however). This can be avoided by employing a domain-based approach. That is, suppose a query with two domains, A and B, hit a PDB sequence with domains A and C. Using a domain-based approach, only small molecules interacting with A are predicted for the query. Using a strictly sequence-based approach, small molecule annotation from C may also be mapped onto domain B of the query, even though B and C are not evolutionarily related.

The construction of protein family or 'domain' databases makes possible the evaluation of the position-specific conservation of residues at binding sites. Conserved domain databases include Protein FAMilies (PFAM) [15] and Simple Modular Architecture Research Tool (SMART) [16], derived from seed alignments and Hidden Markov Models (HMMs), and NCBI's Cluster of Orthologous Genes (COG) [17], obtained by mutual BLAST best hits and appearance in at least three disparate organisms. InterPro [24] attempts to consolidate domain information and remove some of the redundancy between them, assigning a single InterPro identifier to domains that are considered equivalent. The Conserved Domain Database (CDD) from NCBI mixes together domains from PFAM, SMART, COGs as well as their own curated conserved domain families [25]. CDD can be searched using the Reverse Position Specific Basic Local Alignment Search Tool (RPS-BLAST) [26], which compares a given query sequence to the domain sequence profiles for each of the domains in CDD. Instead of finding hits to other proteins, as in standard BLAST, RPS-BLAST returns hits to domain families, providing a prediction of the domain composition of a novel sequence.

In this work, we present the Small Molecule Interaction Database (SMID), a database of interactions between small molecules and CDD protein domains, derived from the data in PDB. Previously, a set of over 23,000 non-redundant protein-small molecule interactions was generated from the PDB using a processing tool producing a structural subset of the Biomolecular Interaction Network Database (BIND) [27] called the molecular modeling database BIND (MMDBBIND) [28]. From this initial structural interaction set, protein domains were identified that associate with one or more small molecules.

SMID presents a unified interface to view detailed small molecule binding sites within conserved families and functional domains while also making available a more detailed view of the underlying protein-small molecule binding sites. The additional layer of abstraction from protein to domain enables SMID to group together all the similar interactions involving a particular domain for easy comparison.

Additionally, we present SMID-BLAST, a tool for small molecule and binding site annotation and prediction. SMID-BLAST acts in a similar manner to *in silico *high throughput screening, yet instead of only searching through a library of small molecules, it uses the structural information in the PDB to extrapolate known small molecule interactions to a protein of interest. In this way, SMID-BLAST can generate a short-list of potential lead-compounds that can be used in further analyses. While it is true that the number of small molecules currently housed in the PDB is smaller than those in certain high throughput libraries, the number and diversity of the small molecule pool in the PDB increases with each new structure added. SMID-BLAST improves over existing methods by using domain annotation to avoid transitive annotation errors and to retrieve more remote homologs, giving additional small molecule hits wherever possible, by providing a confidence score and ranking for each hit, and by filtering out common solvents and other biologically irrelevant molecules.

SMID-BLAST may be used to provide automated annotation on newly sequenced genomes and sequences of unknown function, as small molecule binding can often imply function. We demonstrate through several examples how SMID-BLAST may be used to identify candidate small molecule binding sites on a query sequence of interest that are not found or difficult to identify using similar existing methods, and deduce protein function and evolutionary relationships.

## Utility and discussion

### SMID User Interface

The SMID interface is written in PHP [29] and provides a layer between user queries and the MySQL [30] data tables. SMID may be queried by supplying either a protein GI (of a sequence in PDB), domain identifier, small molecule identifier, PDB ID or SMID ID. All successful queries to SMID return links to individual SMID records. Records contain information pertaining to the protein, domain and small molecule involved in the specific interaction. Interactions involving non-biological contacts with an ion or non-biological small molecules are screened out by default on most queries.

**Figure 1:**
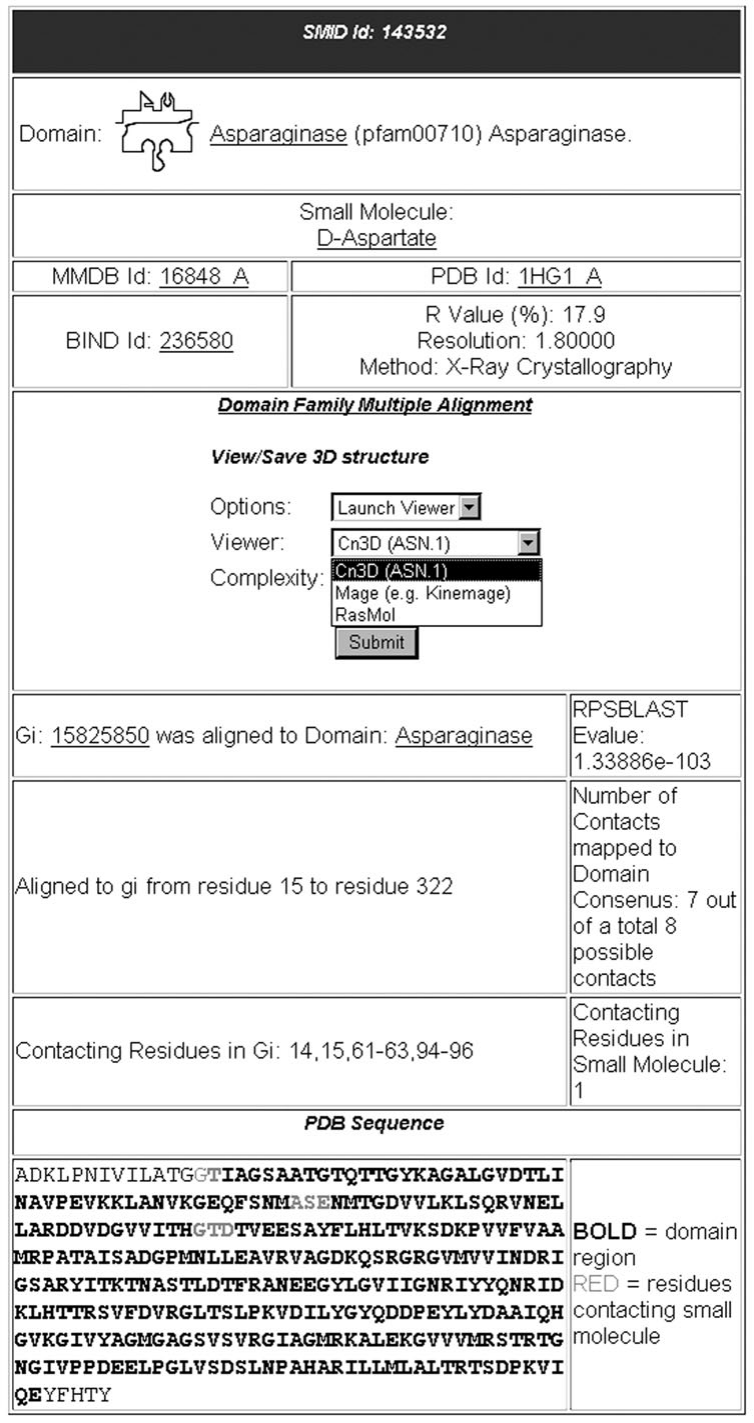
**SMID record as viewed from the SMID web interface**. This record was derived from PDB entry 1HG1, which shows an interaction between an Asparaginase domain (residues 15–322 of chain A, identified by RPS-BLAST with an E-value of 1.34e-103) and D-Aspartate. The GI for 1HG1 chain A is 15825850. For the SMID record shown, seven of the eight residues of the binding site are located within the Asparaginase domain.

**Figure 2:**
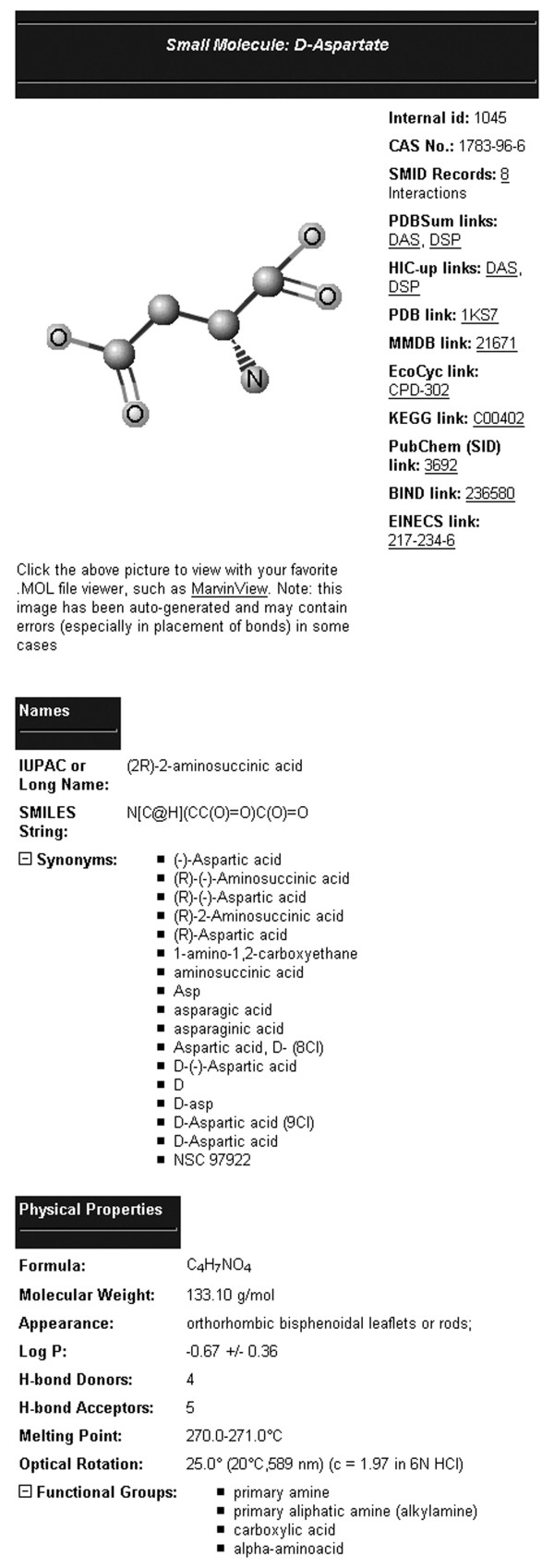
**SMID small molecule information page, as viewed from the SMID web interface**. The small molecule page shown here indicates that 8 SMID records involve the molecule D-Aspartate.

Figure 1 illustrates the structure of a typical SMID record. Clicking on the small molecule link brings up an information page (Figure 2) that contains the molecular structure, links to other databases, common nomenclature and a list of physical properties. An MDL molfile for the small molecule can be obtained by clicking on the ball-and-stick structure. Viewers such as MarvinView [31] or ISISDraw [32] can be used to view files of this type. The database links include other small molecule information pages as well as the parent MMDBBIND interaction. In addition, a link is provided to view all SMID records involving the small molecule of interest. Physical properties listed include molecular formula, weight and functional groups.

**Figure 3:**
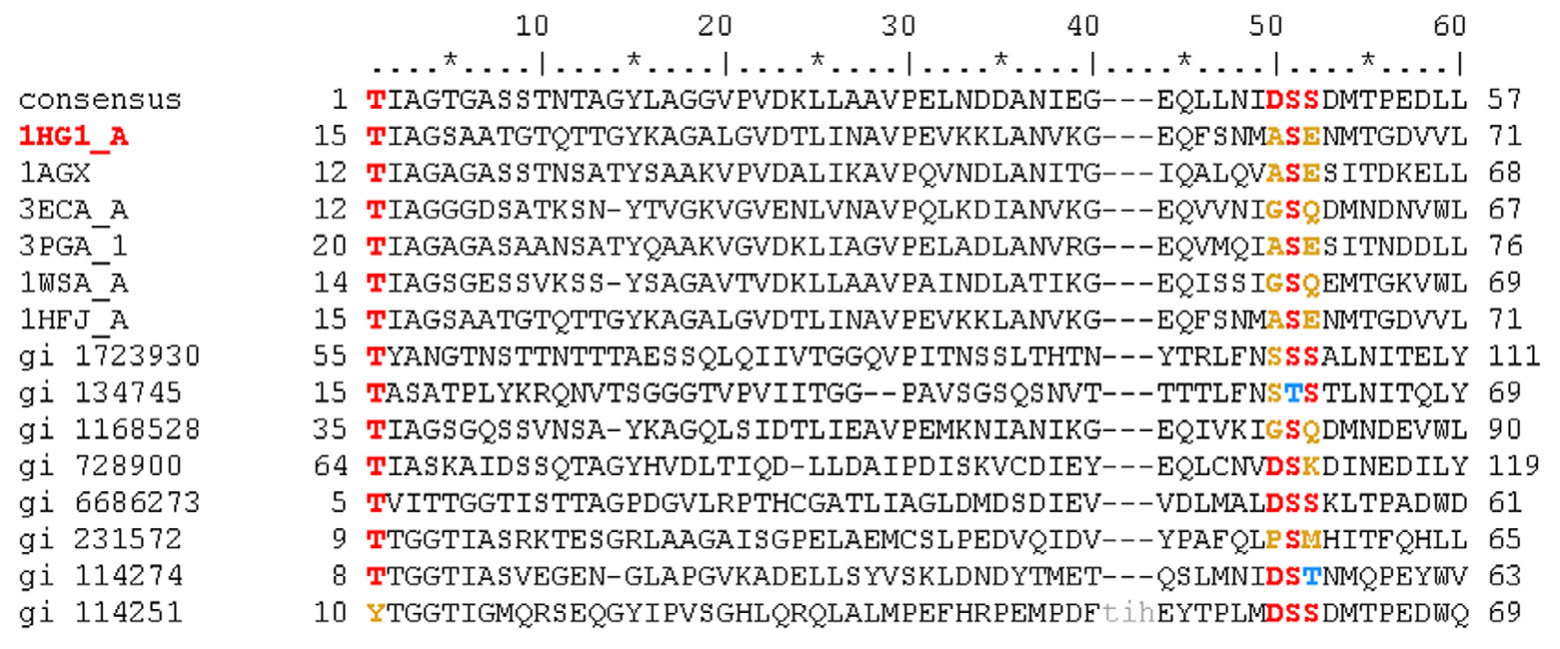
**A CDD domain family multiple alignment**. All sequences from a CDD domain family are listed including the consensus. In addition, the sequence for the PDB protein from which the SMID interaction was derived is included, with its PDB code highlighted in red. Lowercase residues do not align with the consensus and represent insertions or deletions relative to the consensus. Small molecule binding site residues are mapped to the domain family sequences from the parent PDB sequence using the following colour-coding scheme: red for conserved residues, blue for similar residues and yellow for non-conserved residues. In cases where a binding site aligns to a gap in the consensus, conservation cannot be measured and thus no coloured residue is displayed. Note that some binding site residues may be highlighted in addition to those associated with the parent PDB sequence if there are redundant interactions from other PDB files with a similar binding site. This alignment has been truncated for clarity.

The 'Domain Family Multiple Alignment' link provides a complete CDD domain alignment with small molecule binding residues highlighted according to their degree of conservation, as shown in Figure 3. The PDB sequence that is the source of the SMID record is added to the alignment if not already there, and indicated with its name in red.

**Figure 4:**
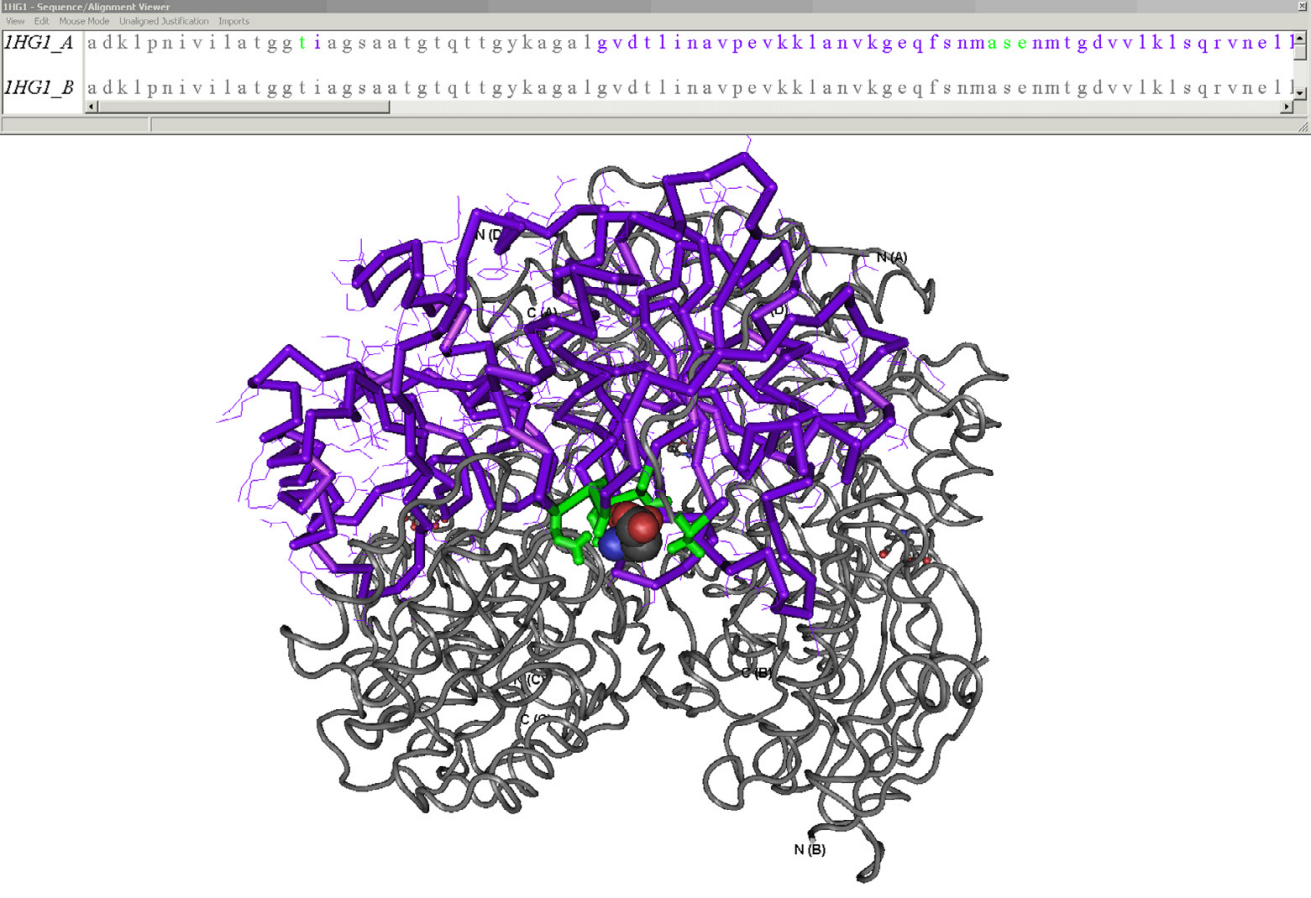
**A 3-D SMID interaction**. The x-ray crystallographic structure of *Erwinia chrysanthemi *L-Asparaginase associating with D-Aspartate (PDB ID: 1HG1), as viewed by Cn3D. The structure was annotated by SMID to highlight the domain residues (purple), domain residues contacting the D-Aspartate molecule (green) and the non-domain residues (grey). The D-Aspartate small molecule ligand is shown in space-fill format. The sequence/alignment viewer provides sequences for all chains found in the PDB record. For the sequence involved in the small molecule interaction, residues are colour-coded using the same scheme seen in the structural model.

The 'View/Save 3D structure' section provides a means to view the SMID interaction using Cn3D or RasMol. When viewed in Cn3D (Figure 4), domain residues are highlighted in purple while non-domain residues are in grey. Residues within the domain contacting the small molecule are highlighted in green.

The 'RPS-BLAST Evalue' in Figure 1 serves as an indicator of confidence that a particular CDD domain (e.g. Asparaginase), exists in a PDB protein. The bottom section of a SMID record describes the location of both the CDD domain and the small molecule binding residues in the parent PDB protein sequence from which the record is derived. This is depicted both numerically and graphically. For the latter, the PDB protein sequence is provided with domain residues in bold and binding site residues in red. Finally, the fraction of small molecule binding site residues found within the domain region is provided, as sometimes ligand residues lie outside the range of the CDD domain definition.

Querying SMID using either domain or small-molecule designations provides the option of viewing either a non-redundant or redundant set of records. Redundant SMID records are clustered according to the rules outlined in Methods. Searching with either a PDB protein GI or domain identifier designation returns a listing of associating small molecules along with links to the corresponding SMID records. A domain query can involve a CDD Position Specific Scoring Matrix (PSSM) ID, Interpro ID, Pfam/Smart ID or a descriptive keyword. To assist in querying, a link is provided on the SMID website that displays all domains in SMID, along with their PSSM ID, short name and description. Querying with a small molecule identifier returns a listing of all associating domains. Where applicable, an Interpro [24] ID is provided to assist in identifying redundant domain hits. A small molecule query may be entered as a PDB HET code (e.g. HEM for heme) or as a case-insensitive keyword. As with domains, a link is provided which lists all the small molecules in SMID. The list includes the HET name 3-letter code or other short label, which serves as a link to the corresponding small molecule info page (Figure 2), and the full compound name. Querying with a PDB ID returns a listing of small molecules binding any protein chain in the PDB structure record. Lastly, individual SMID records may be obtained by searching with SMID IDs. It should be noted that SMID IDs are unstable and may change in later versions of SMID. Therefore they should not be used to keep track of a particular SMID interaction. The PDB ID or BIND ID, which can be found in a SMID record, are much more suitable for this purpose.

### SMID-BLAST

To enable users to identify putative small-molecule binding sites in proteins for which a crystal-structure has not yet been determined, the SMID interface includes the SMID-BLAST web tool. SMID-BLAST executes NCBI's RPS-BLAST algorithm on a query protein sequence, identifying structural domains from CDD that have small molecules bound, as found in SMID. A SMID-BLAST query may take the form of an amino acid FASTA formatted sequence, protein GI, accession from any common sequence database or PDB chain. As with RPS-BLAST, the user can configure the Expect value cutoff, search mode and use of a low complexity filter. In addition, the user is able filter out SMID interactions involving non-biological ion contacts or non-biological small molecules, as detailed in Methods.

SMID-BLAST hits are represented as a gapped local alignment between the query protein and a CDD domain with small-molecule binding sites mapped to the query from all small molecule interactions in SMID involving that domain. For SMID records that are redundant, the union of the binding sites of all members of the redundant group is used when mapping the binding site to the query. Binding site residues are colour-coded based on the total number of non-redundant interactions they participate in. Each SMID-BLAST hit also includes a table summarizing all putative binding small molecules along with their binding sites in the query and the number of redundant SMID records supporting the interaction. Clicking on the binding sites displays a domain family multiple alignment similar to that found in a SMID record (Figure 3) but including the SMID-BLAST query sequence. The PDB sequence from which the SMID record is derived is also included in the alignment, highlighted in red. In addition, for redundant interactions, highlighted regions correspond to the union of the domain-binding residues from all members of the redundant group.

SMID-BLAST also calculates a heuristic ligand score as a confidence measure to indicate the likelihood that the query really binds the small molecule at the stated binding site. The initial ligand score is computed for each putative binding site of each small molecule hit. After all the hits have been processed, a final small molecule summary table for the query is provided. Where possible, similar binding sites for the same small molecule from different domain hits are combined into a single binding site by taking the union of the residue numbers. This often results in binding sites larger than any one single binding site, and reflects the variability in the location of the binding residues between different examples of the domain-small molecule interaction or uncertainties in the domain multiple alignment. The table column 'hits' provides the number of SMID-BLAST hits that were averaged and combined to make each binding site. Molecules in the summary table are sorted by the final ligand score, which is the average of the initial ligand score for each similar binding site that was merged together to form the summary binding site, multiplied by the binding site occupancy.

The final summary table provides at-a-glance the small molecules most likely to bind the query sequence, as well as the most likely binding site(s) for them. It has been determined empirically that hits with a final ligand score above 50 tend to be true interactors, and is thus the recommended threshold value for annotating binding sites. Values less than 50 may be considered possible interactors for predictive purposes, and appear greyed out. As this score is a function of other well-known sequence scores, the threshold can be easily understood as corresponding to the default RPS-BLAST E-value cutoff of 0.01 together with a binding site residue identity of 30%, and a relative entropy value of 1 for the binding site residues in the context of the conserved domain (see Methods). The score does not simply recapitulate the RPS-BLAST E-value scores retrieved. A more thorough statistical analysis of the ligand score and its relation to significance will be carried out in future work.

A command line version of the SMID-BLAST tool is provided that does not require access to the web-based system, for high-throughput sequence annotation applications. The tool will accept a file of FASTA-formatted protein sequences as input and output an ASN.1 formatted file containing the final summary table data for each input sequence. This can then be read in by NCBI's Sequin program [33] for further analysis and annotation. The command-line SMID-BLAST will automatically use Sequin to convert the output file to GenPept format as well. Command-line SMID-BLAST requires a license and interested parties should contact the authors.

### SMID-BLAST validation

Although it is well known that some proteins may bind several ligands in the same binding site, the solved protein structure will only show a single ligand in a particular binding site. SMID-BLAST may predict several possible ligands for a site, and thus it is difficult to assess whether a small molecule predicted to bind that is not the exact small molecule is in fact a plausible alternative/unknown or whether it is a *bona fide *false positive. The validation approach consisted of determining how many protein small molecule interactions could be predicted from the most recent entries in the PDB as well as determining the binding site coverage from predicted interactions. The experimental data set for the validation studies consisted of 793 small-molecule interactions from 581 non-redundant protein chains (the remainder of the 2379 newly released chains did not have interactions, or were redundant with existing interactions). SMID-BLAST correctly predicted the ligand in 472 (60%) of the experimentally determined interactions, of which 315 (66%) obtained the best final ligand score. For correct molecule predictions, 344 (72%) of the predictions had greater than 80% of the binding sites correctly identified.

**Figure 5:**
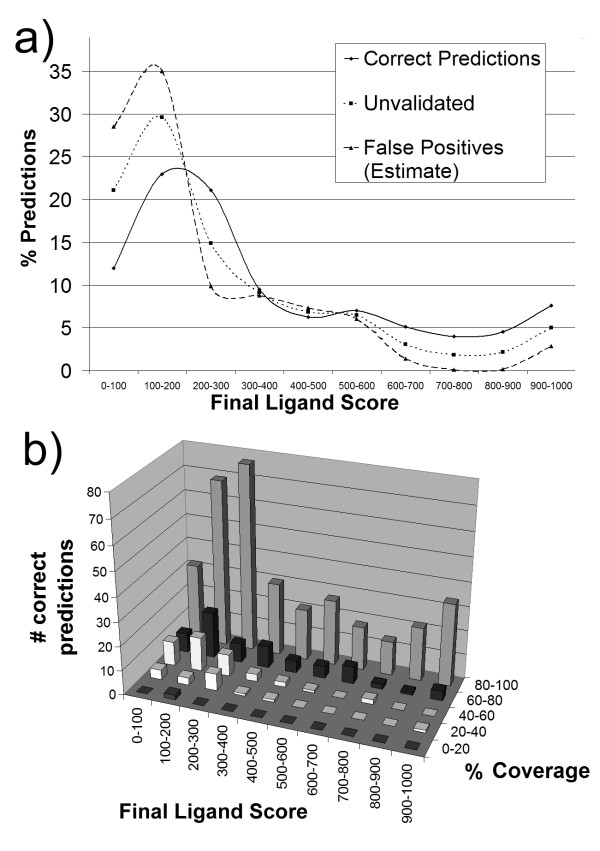
**SMID-BLAST validation final ligand score distributions**. a) Distribution of predictions in the validation set as a function of final ligand score. The solid line represents percent correct predictions, while dotted line represents predictions that were not observed in the PDB validation set; these latter interactions are comprised of both false positives, and true positives that simply have not been observed yet. For example, 12% of correct predictions had a final ligand score below 100, while 21% of unvalidated predictions had a final ligand score below 100. The dashed line represents an estimate of the distribution of final ligand scores for false positives as outlined in the text. b) Coverage as a function of final ligand score, for the predictions which were observed in the PDB validation set. Coverage is defined as the percent of true binding site residues which were included in the predicted binding site.

Figure 5a shows the relation between final ligand score and correct prediction for this validation experiment. We find that, in general, unvalidated predictions are weighted more towards lower ligand scores under 200, compared to correct predictions. Note that not all of the unvalidated hits are false positives, we simply do not have evidence supporting them, and so they represent a worst case number of false positives. The fraction of true positives with ligand score above 200 is substantially increased over the fraction of unvalidated predictions in this range.

Further, let us suppose that the 'unvalidated' interaction curve U in Figure 5a is made up of two components – one from correct predictions C which were simply not observed and one from false positives F, where U, C and F are all normalized to have the same area under the curve. If we assume that curve C has the same shape as the curve for the correct observed predictions, and the fraction of correct predictions in the unvalidated curve is α, then U = αC + (1 - α)F. If we further assume that at score > 700, all the unvalidated predictions are correct and set α such that F ≈ 0 (i.e. no false positives at score > 700) we obtain α = 0.45. This value provides a rough estimate that at most 45% of the unvalidated interactions may be true positives across the entire score range. This is important, because even in the highest final ligand score bin of 900–1000 the number of unvalidated predictions is about 9 times the number of validated ones. Recall, that it would take a large number of co-crystal structures to validate these predictions.

Figure 5b shows, for the correct predictions, the relationship between final ligand score and percentage coverage of binding sites predicted. Here we see that most of the correct ligands have excellent binding site coverage (80–100%), and this increases with ligand score. The examples with poor binding site coverage (less than 60%) are almost exclusively limited to ligand scores below 300. Taken together, these provide support that the final ligand score is a discriminating factor in 1) selecting the correct ligand and 2) realizing the best possible binding site coverage.

As an illustrative example, we considered the interaction between the protein diaminopimelate dicarboxylase (GI: 29726280, MMDB: 32196, PDB:1HKV chain A) from *Mycobacteria tuberculosis *and the pyridoxal-5'-phosphate (PLP) ligand. A SMID-BLAST prediction for this sequence identified the PLP ligand using 9 different PDB chains, of which the *Escherichia coli *diaminopimelate decarboxylase protein (GI: 39654106, MMDB: 25220, PDB: 1KNW chain A) had 27% sequence identity, the highest of all 9 PDB chains. The SMID-BLAST prediction correctly predicted 11 of the 12 experimentally defined binding site residues. The prediction also included 6 additional residues sufficiently close to the observed binding site that they could have been included if the MMDBBIND algorithm's threshold distance cutoff for identifying residue interactions had been increased.

### SMID-BLAST examples

**Figure 6:**
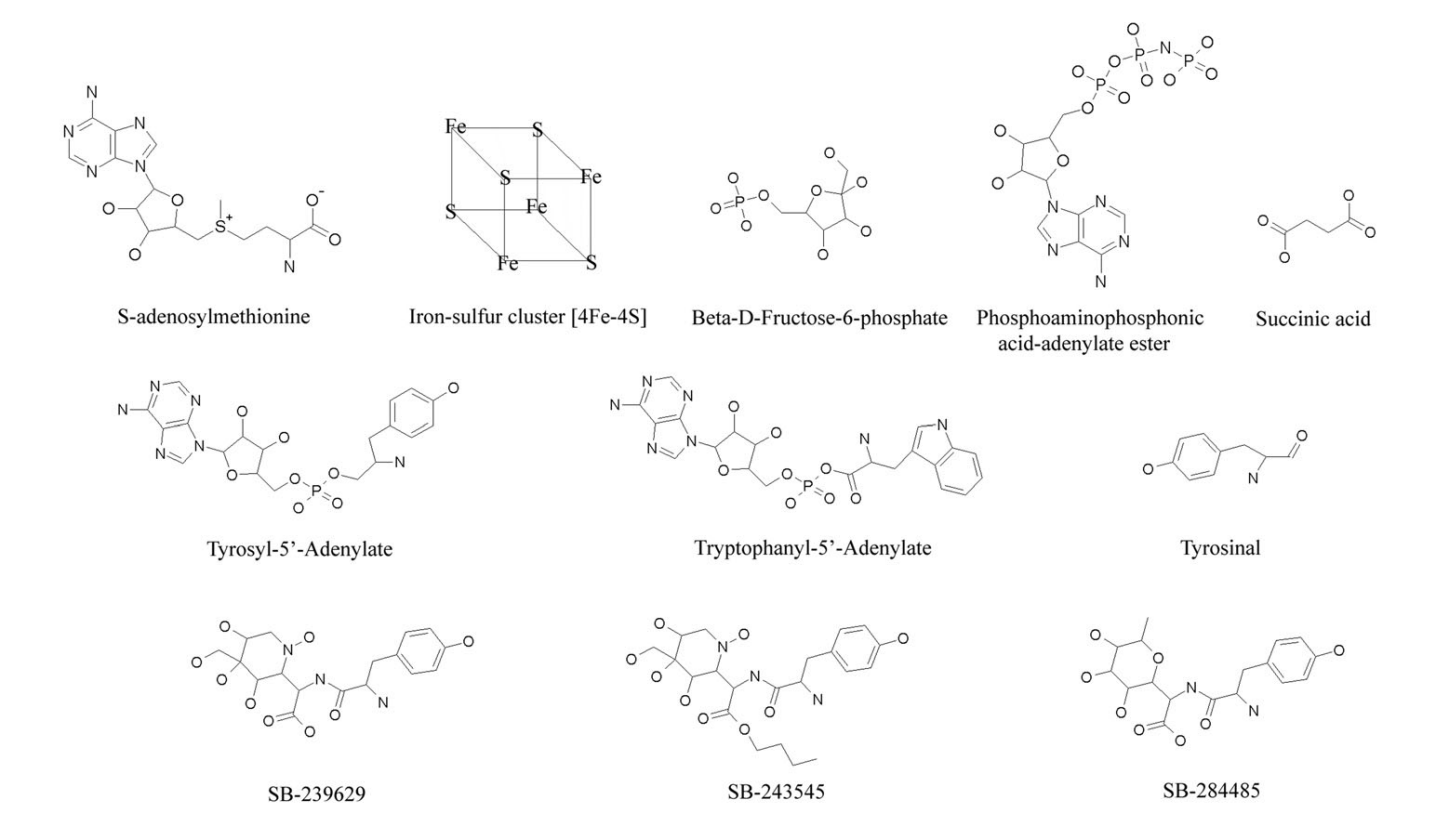
**Selected chemical structures**. Chemical structures of selected SMID-BLAST small molecule hits from query proteins MiaB, Phosphoglycerate Mutase, TrpRS and TyrRS.

The following examples illustrate some of the possible uses for SMID-BLAST, and compare its output to that of Relibase and SAS. Such examples illustrate the ability of SMID-BLAST to accurately extrapolate from protein-small molecule interactions found in the PDB. Figure 6 provides the structures for a selected set of SMID-BLAST small molecule hits mentioned below. The default SMID-BLAST options were utilized for all queries.

#### Burkholderia pseudomallei K96243 tRNA thiotransferase

The MiaB protein is a tRNA thiotransferase enzyme that is involved in the post-translational modification of tRNAs. Specifically, the MiaB protein has been shown to be involved in the thiolation and methylation steps leading to the synthesis of the 2-methylthio-N^6^-isopentenyl-adenosine (ms^2^i^6^A) modified tRNA nucleoside [34,35]. MiaB is an example of a Radical SAM enzyme, a group of proteins that participate in numerous biosynthetic pathways [36]. All members of this group contain an iron-sulfur cluster ([4Fe-4S]) coordinated by S-adenosylmethionine (SAM) and three closely spaced cysteine residues. The cysteine residues are part of a conserved triad motif, Cys-XXX-Cys-XX-Cys, found in all Radical SAM enzymes. The putative MiaB protein (GI 53718317) from *Burkholderia pseudomallei *(*B. pseudomallei*) shares a high degree of sequence similarity to MiaB proteins found in other bacterial organisms and possesses the highly conserved cysteine triad motif. The MiaB protein was chosen as a query to SMID-BLAST due to the fact that its crystal structure has yet to be solved. Furthermore, a BLAST search of the *B. pseudomallei *MiaB protein against the PDB returns only very weak hits. These factors make this protein an excellent query to highlight the predictive capabilities of the SMID-BLAST algorithm.

SMID-BLAST identifies one putative small molecule binding site (Table 1). Both of the hits to this site, [4Fe-4S] (F4S, score = 181) and SAM (score = 108), had final ligand scores above the cutoff value and are the known ligands of the MiaB protein. The predicted binding site for F4S on MiaB was mapped from sites extracted from crystallized structures of the *Escherichia coli *HemN (PDB 1OLT) and Biotin Synthase (PDB 1R30) proteins, as well as the *Staphylococcus aureus *protein MoaA (PDB 1TV7 and 1TV8). All of these proteins are members of the Radical SAM enzyme group. The cysteine triad motif in the *B. pseudomallei *MiaB protein occurs at residues 157–164 (**C**SKY**C**SY**C**), which overlaps with the predicted F4S binding site provided by SMID-BLAST (Table 1). Most importantly, all three cysteine residues in the triad are predicted to be associated with F4S.

The SMID-BLAST binding site for SAM was mapped from the same four PDB structures as F4S. It has been shown that SAM must be placed in the immediate vicinity of the F4S in order to mediate catalysis [37]. For example, a distance of 2.7 Å was identified between SAM and an iron atom from the F4S cluster in the Radical SAM enzyme lysine 2,3-amino-mutase [38]. SMID-BLAST correctly predicts this close association by placing SAM and F4S in the same binding cleft.

**Figure 7:**
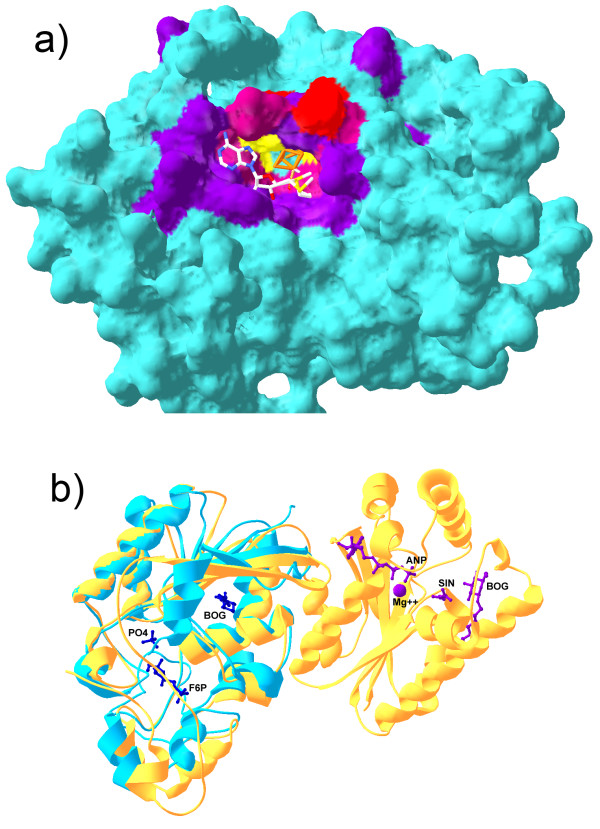
**Binding sites predicted by SMID-BLAST**. a) Shown is a comparative model of the predicted Elp3 domain of MiaB. The iron-sulfur cluster (orange) and SAM (CPK stick model) have had their co-ordinates transferred from the modelling template, PDB 1OLT chain A to illustrate how they might bind. The predicted Fe-S binding site residues are indicated in red, the predicted SAM binding residues are shown in purple, and the three cysteine residues which interact with the Fe-S cluster are indicated in yellow. A mixture of red and purple was used for residues common to both binding sites. b) Structural alignment of PDB 1RII chain A (phosphoglycerate mutase from *M. tuberculosis*, blue) and 2BIF chain A (6-phosphopructo-2-kinase/fructose-2,6-bisphosphatase from R*attus norvegicus*, yellow). The small molecules from 2BIF are also shown along with their PDB short labels. Purple molecules associate with the N-terminal domain of 2BIF chain A, while blue molecules associate with the C-temrinal domain. Note that BOG was part of the crystallization buffer in this example. Structures were aligned with Swiss PDBViewer.

To obtain a closer look at the predicted binding site, the MiaB sequence was submitted to the SAM-T02 [39,40] fold recognition server, to obtain a template and sequence alignment for modeling. The top hit was PDB 1OLT_A with a template E-value of 3.4e-24. Resdiues 121–404 of MiaB aligned to 50–286 of the template, so this portion was modeled using SwissModel [41]. The F4S and SAM from 1OLT were simply copied with the same co-ordinates into the model. As seen in Figure 7a, the binding site rapidly predicted by SMID matches well with the binding pocket on the model, and includes the three critical Cys residues, with only two residues which are clearly not part of the binding site.

For comparison purposes, Relibase and SAS were also queried with the *B. pseudomallei *MiaB sequence. Relibase failed to identify any known MiaB ligands. The small molecules it did return were various ions, DNA strands and nucleotide phosphates, but all PDB hits had a percentage sequence identity score less than 25%, and thus do not have a high degree of confidence associated with them. The SAS service also did not return any high confidence structure hits, as the best E-value obtained was 0.2. While SAS returned a hit to the SAM radical protein MoaA (1TV8) co-crystallized with SAM and an [4Fe-4S] cluster, the E-value was very poor, 8 (28.8% identity). It should also be noted that SAS provided a number of small molecule hits, such as sulfate and dithiothreitol that are not biologically significant ligands to MiaB.

#### Mycobacterium tuberculosis CDC1551 Phosphoglycerate Mutase

Phosphoglycerate mutase (PGM) is a ubiquitous enzyme that is primarily involved in the interconversion of 3-phosphoglycerate (3PG) and 2-phosphoglycerate (2PG) in both glycolysis and gluconeogenesis [42]. Two types of PGMs have been identified, one that is dependent on the cofactor 2,3-bisphosphoglyceric acid for activity (dPGM) and one that is not dependent on a cofactor (iPGM). The PGM from *Mycobacterium tuberculosis *CDC1551 (*M. tuberculosis*) is a cofactor-dependent enzyme whose three-dimensional structure has recently been solved [43]. While this structure was not co-crystallized with any known dPGM ligands, other dPGM structures exist in the PDB with ligands such as 3PG (3PGM) and vanadate (1E59). Thus, this particular query is not meant to highlight the predictive powers of SMID-BLAST. Rather, the *M. tuberculosis *dPGM query illustrates the importance of using protein domain information to avoid transitive annotation errors in ligand assignment as will be seen below.

Querying Relibase with the *M. tuberculosis *dPGM sequence (GI 15839880) returns hits including dPGM structures such as 1BQ4, 3PGM and 1E59, as well as structures of proteins that are homologous to dPGM, such as rat fructose-2,6-bisphosphatase (F26BPase) in 2BIF. A strong evolutionary relationship has been established between dPGM and F26BPase using both structural and sequence analysis [44-46]. In the SCOP database [47], both dPGM and F26Bpase cluster together at the 'family' level. In addition, both the *M. tuberculosis *dPGM and the rat F26Bpase contain the same domain, the phosphoglycerate mutase family domain (pfam00300). However, while dPGM is a single domain protein, the F26BPase protein in 2BIF consists of two domains: a 6-phosphofructo-2-kinase domain (pfam01591) at the N-terminus and pfam00300 at the C-terminus.

Figure 7b shows a structural alignment between the query protein, PDB 1RII chain A, and 2BIF, chain A. The crystal structure of F26BPase in 2BIF depicts the N-terminal domain (on the right of Figure 7b) associating with magnesium, phosphoaminophosphonic acid-adenylate ester (ANP) and succinic acid (SIN) while the C-terminal domain associates with beta D-fructose-6-phosphate (B-D-Fructose-6-P). Thus, while B-D-Fructose-6-P would be considered a possible ligand for dPGM, which shares the phosphoglycerate mutase domain, all small molecules associating with the N-terminal domain of F26Bpase would not be possible ligands since dPGM lacks this domain. Relibase, by virtue of dPGM and F26BPase sharing a significant degree of global sequence identity makes a significant transitive error by listing all ligands of F26BPase as possible ligands to dPGM.

In contrast, SMID-BLAST does not recognize magnesium, ANP or SIN as small molecule ligands to the *M. tuberculosis *dPGM protein (Table 2). The only small molecule SMID-BLAST hit from structure 2BIF is B-D-Fructose-6-P, which associates with the pfam00300 domain found in both dPGM and F26Bpase. The BOG crystallization buffer molecule found by Relibase was explicitly filtered out by our non-biological small molecule filter (see Methods). While B-D-Fructose-6-P does not appear to associate with dPGM in vivo, the two could possibly associate in vitro given the fact that a number of key catalytic residues in the pfam00300 domain of F26Bpase are conserved in the dPGM protein [44]. SMID-BLAST also identifies known dPGM ligands such as the substrate 3PG and inhibitor molecules such as inositol hexaphosphate (IP6) and benzene hexacarboxylate [48] as well as tetrametavanadate (VO_3_) [49].

#### Tyrosyl and Tryptophanyl tRNA synthetases

Tryptophanyl tRNA synthetase (TrpRS) and Tyrosyl tRNA synthetase (TyrRS) are known to share a similar structural core and are paralogous enzymes. This is illustrated in CDD with the TyrRS_core family (cd00805), TrpRS_core family (cd00806) and their parent family Tyr_Trp_RS_core (cd00395). It is likely that Tyr vs. Trp discrimination developed late in the evolutionary origin of the genetic code [50,51] compared to other tRNA synthetases, and both may have originated from a hypothetical non-specific Phe *-*Tyr(Trp)RS progenitor synthetase. Thus the pair serve as a good test for SMID-BLAST's sensitivity in identifying the true targets of enzymes.

TrpRS aminoacylates tRNA with the activated intermediate tryptophanyl adenylate (TYM) while for TyrRS it is tyrosyl adenylate (TYA). While both synthetases may be expected to have some affinity for both these molecules, they should show preferential binding to their cognate substrate molecules (i.e. TrpRS to TYM, TyrRS to TYA). To test this, 82 TrpRS sequences [see Additional file 1], and 83 TyrRS sequences [see Additional file 2] from a diverse set of organisms from all kingdoms of life, were submitted to SMID-BLAST. The top scoring two ligands were recorded for each sequence, and the results are summarized in Table 3.

Since all tRNA synthetases bind ATP [52], we looked at the top hit ignoring ATP and magnesium ions. The results indicate that the TrpRS enzymes almost all had a higher final score for the expected substrate, TYM, than for TYA and its analogues. The only exceptions were *Methanosarcina acetivorans *C2A and *Picrophilus torridus *DSM 9790, both of which identified tyrosinol, a tyrosine analogue, as the most likely ligand.

TyrRS had TYA in first place only 37 of 83 times. Another 32 sequences had one of three potent bacterial TyrRS inhibitors, discovered through high-throughput screening of natural products [53] as their top hits. Of these 32, 23 were bacterial TyrRS's. Of the remaining TyrRS sequences, thirteen hit TYM, the cognate substrate of TrpRS – three eukaryotic and ten archaeal extremophiles. Lastly, *Methanothermobacter thermautotrophicus *str. Delta H found D-tyrosine as the strongest hit.

SMID-BLAST was able to identify the preference of TrpRS for TYM over TYA and related analogues 80 out of 82 times in a wide variety of species. It did not perform as well on TyrRS, but still gave remarkable results, since the three TyrRS inhibitors in PDB would be expected to have higher affinity than TYA in many bacterial species [53]. Only 13 out of 83 sequences were misassigned as being more favourable towards TYM binding.

Upon inspection of our heuristic score, the main contribution to the 13 mis-ranked TyrRS binders was from the occupancy component of the final ligand score. Given two molecules of different size, and a binding site large enough to accommodate either one (as is the case here), the larger one is more likely to better fill the binding site and generate a higher occupancy score. Thus in 13 cases this difference is enough to push the final ligand score for TYM above that for the smaller TYA. To demonstrate this, we repeated the computation without the occupancy component, also shown in Table 3, and indeed all the hits to the TyrRS's were TYA, Tyrosinal, D-tyrosine, or SB-239629 (TyrRS inhibitor). However, without the occupancy component of the score, the TrpRS small molecule hit list becomes intermingled with smaller molecules like L-tryptophan, D-tyrosine and SB-239629 as well. Clearly the binding site occupancy plays an important role in binding site scoring, and this component is critical to the performance of the overall final ligand score in ranking small molecule hits.

These observations demonstrate that while the final ligand score is by no means perfect and may not correspond directly to binding constants, it does give some reliable indication of what small molecules are most likely to bind the target. This information can be used to prioritize further experiments and tests. The fact that the TrpRS proteins generally only hit Trp and its analogues, and not Tyr, while some TyrRS query sequences seem to hit Trp and its analogues, may indicate that TrpRS is more specific. Hence TyrRS may be closer to the non-specific Phe-Tyr(Trp) precursor enzyme, while TrpRS has evolved sufficiently away to uniquely identify its larger, bulkier target.

## Conclusion

SMID provides an extremely useful extrapolation of the small molecule interaction information implicitly stored in the PDB database. By generalizing from protein-ligand interactions to domain-ligand interactions, SMID is able to cluster similar interactions and detect subtle binding patterns that would not otherwise be obvious. Using SMID-BLAST, likely small molecule targets can be predicted for an arbitrary protein sequence, with the only limitation being that the small molecule must exist in the PDB in order to be predicted. There are presently over 5000 unique small molecules represented in the PDB, many being drug-like molecules, and so SMID-BLAST could be used at least as a starting point to suggest what small molecules may bind to a protein of interest. More importantly, SMID-BLAST can suggest where specific small molecules are likely to bind the protein.

The SMID-BLAST validation results, coupled with the specific examples listed in the results section, illustrate that SMID-BLAST has a high degree of accuracy in terms of both identifying a small molecule ligand and predicting the binding site residue positions for a query protein. This level of accuracy will only increase as more protein structures are deposited into the PDB and hence more interactions are computed for SMID. SMID-BLAST on average overestimates both the number of possible small molecule ligands for a given binding site as well as the number of residues in a binding site. This latter observation reflects the variability in the binding site between different examples of the domain-small molecule interaction or uncertainties in the domain multiple alignment.

Regarding the overestimation of binding site ligands, it may be the case that some of the extra ligands predicted for a given binding site are true interactants but have simply not been crystallized with the protein of interest. The observed overestimation of binding site ligands may also involve the identification of one or more false ligands. For example, a small molecule that is known to bind to a domain in one protein may not bind to the same domain in a different protein. This could result from structural variations between the two domains, point mutations or variations in total protein structure. While the final ligand score does not correlate perfectly with binding affinity, ligands such as synthetic transition state analogues will often have a higher score. For example, the top scoring SMID-BLAST hit for 32 out of 83 TyrRS enzymes analyzed was an inhibitor compound and not the endogenous substrate TYA (Table 3). In some cases, the existence of multiple false ligand predictions for a protein can result in lower final ligand scores for true interactants. This can happen, for instance, if a false ligand prediction fills more of the predicted binding site than the true ligand(s), thus giving it a higher occupancy score. The PDB identity score may also be higher for the false versus the true small molecule ligand. The existence of un-validated ligand predictions for a given query protein does not discredit the usefulness of the final ligand score for identifying small molecule interactants however. SMID-BLAST can save researchers a great deal of time by providing a short-list of probable ligands, ranked by a confidence measure, that can be verified experimentally.

Possible uses of SMID-BLAST include prioritizing drug docking experiments, selecting structure templates for homology modeling, or annotating complete genomes. Computational methods such as docking could be used to establish whether the top scoring SMID-BLAST hits really do bind at the sites indicated. The number of molecules that would have to be screened would be very small, allowing detailed, more complex docking algorithms to be used for more accurate results. The small molecule binding sites predicted by SMID-BLAST would also serve as an efficient means of selecting a small pool of drugs for experimental analysis in the lab.

In homology modeling, selecting the best structure template is critical for generating an accurate protein structure prediction. Since small molecule binding sites are generally highly conserved among members of a given protein family, structure templates can be chosen that possess a high degree of sequence similarity with the SMID-BLAST predicted binding site residues. Of course, any knowledge about which small molecules the protein being modelled interacts with could be used to improve discrimination between templates even further.

Finally, the command-line SMID-BLAST tool (see Methods) can be used to rapidly annotate protein sets from entire genomes. The tool will take an arbitrary set of sequences and add annotation, in GenPept format, indicating what small molecules are predicted to bind, with the final ligand score provided as a confidence measure. Additionally the PDB and BIND records the prediction is based on will be provided as a cross-reference automatically. This can provide a wealth of information to researchers eager to investigate new proteins and can serve to direct experimentation.

We hope that in the future SMID will serve as a useful resource for interaction prediction, and annotation of new protein sequences.

## Construction and content

### MMDBBIND database

The source for SMID was the small molecule division (3DSM) of MMDBBIND [28], a database of high quality protein-small molecule interactions generated from NCBI's MMDB [54]. The MMDB currently houses over 27,000 molecular structures from the PDB. Protein-small molecule interactions were identified using an interatomic distance cutoff of the van der Waals radii [55,56] plus 0.5Å. For most atom types this results in a distance cutoff of 3.5–4.0Å, typical for a hydrogen bond [57]. A subsequent filtering process identifies and tags those interactions in MMDBBIND which involve i) a single atomic contact ii) a protein with an unknown sequence (i.e. only alpha carbon trace is present in the structure) iii) a biologically irrelevant small molecule (see below) iv) one or more false contacts with a biologically relevant ion. The former two are removed altogether from the database, and will not be considered further.

Protein structures in the PDB often contain agents, such as buffers, salts, detergents, solvents and ions, which aid in the purification and/or crystallization processes, but are not involved in the biological function of the protein [58]. Biologically irrelevant molecules were determined manually to form a curated subset of small molecules consisting of known buffers, detergents, solvents and non-biological ions [see Additional file 3]. A complete list can be viewed through the SMID web interface [59]. It should be noted however that a number of interactions involving a protein and a biologically relevant ion are the result of crystal packing artefacts. For example, two of the three calcium ions in 1OMD (Oncomodulin) [60] are bound to the CD and EF loops while the third calcium ion is found on the surface. This latter calcium ion is co-ordinated to oxygens belonging to three different protein molecules and helps stabilize the crystal structure. Crystal artefacts like this one, involving a potentially biologically relevant ion, were removed using a trained Support Vector Machine (SVM) [61,62].

The ion filtering SVM was trained on 11 attributes as follows. Burial was defined as the fraction buried surface area (inaccessible to water) of the ion compared with the surface area of a perfect sphere of the same radius. N, O, S and total neighbours count the number of contacting nitrogen, oxygen, sulphur, and total atoms, respectively, using a cutoff distance of the sum of the van der Waals radii plus 0.5Å. Only sulphur atoms from Cys residues are included as S neighbours, since these are the only ones likely to form associations with metals. For the remaining six training attributes, the number of atoms within several distance bins from the ion were recorded: from 0–5Å, 5–6Å, 6–7Å, 7–8Å, 8–9Å and 9–10Å. Training sets ranging in size from 20 to 100 examples, of both biological and non-biological ions in crystal structures, were created by human experts for bromine, calcium, chloride, cobalt, copper, iron, potassium, magnesium, manganese, molybdenum, sodium, nickel and zinc. All ions used a radial kernel with gamma and C chosen to produce optimal 10-fold cross-validation, except potassium which used a polynomial kernel of degree 5. All had epsilon = 0.01. The filter removed a total of 12,582 interactions with these ions out of a total of 35,165 in the MMDBBIND set. Additionally, 3,652 interactions with other non-biological ions were removed. Training sets will be made available upon request.

Aside from protein-small molecule interactions, MMDBBIND also contains interactions between protein and small polymers. These include cyclic peptides (antibiotics), oligopeptides consisting of mostly non-standard amino acids, and branched and cyclic polysaccharides, all of which are found in the PDB. For the purposes of this work, these are all considered to be 'small molecules' as well. It is important to note that special care has been taken to include only true polymers. For example, because sugar monomers can attach in several different ways, and PDB files do not always contain explicit or correct bond information [63], connectivity was inferred based on the rules of chemistry. Similarly, some crystal structures may contain multiple conformations of the same molecule (sometimes with different occupancies). The molecules hexane and octane in the structure 1CWQ is an example of this. Special checks were done to avoid making a dimer molecule out of such cases.

MMDBBIND protein-small molecule records serve as a set of fully annotated interactions (3DSM division) in the BIND database [27], and can be accessed through the BIND interface [64] or downloaded [65] through FTP. The BIND records contain short labels, aliases and descriptions for both interactants from such database sources as Entrez Gene, PDB and PubChem. In addition, the records include a listing of small molecule contacting residues in the protein along with a structural model, which can be viewed using Cn3D [66,67]. Cn3D will automatically highlight binding residues in the protein.

### Generation of SMID records

**Figure 8:**
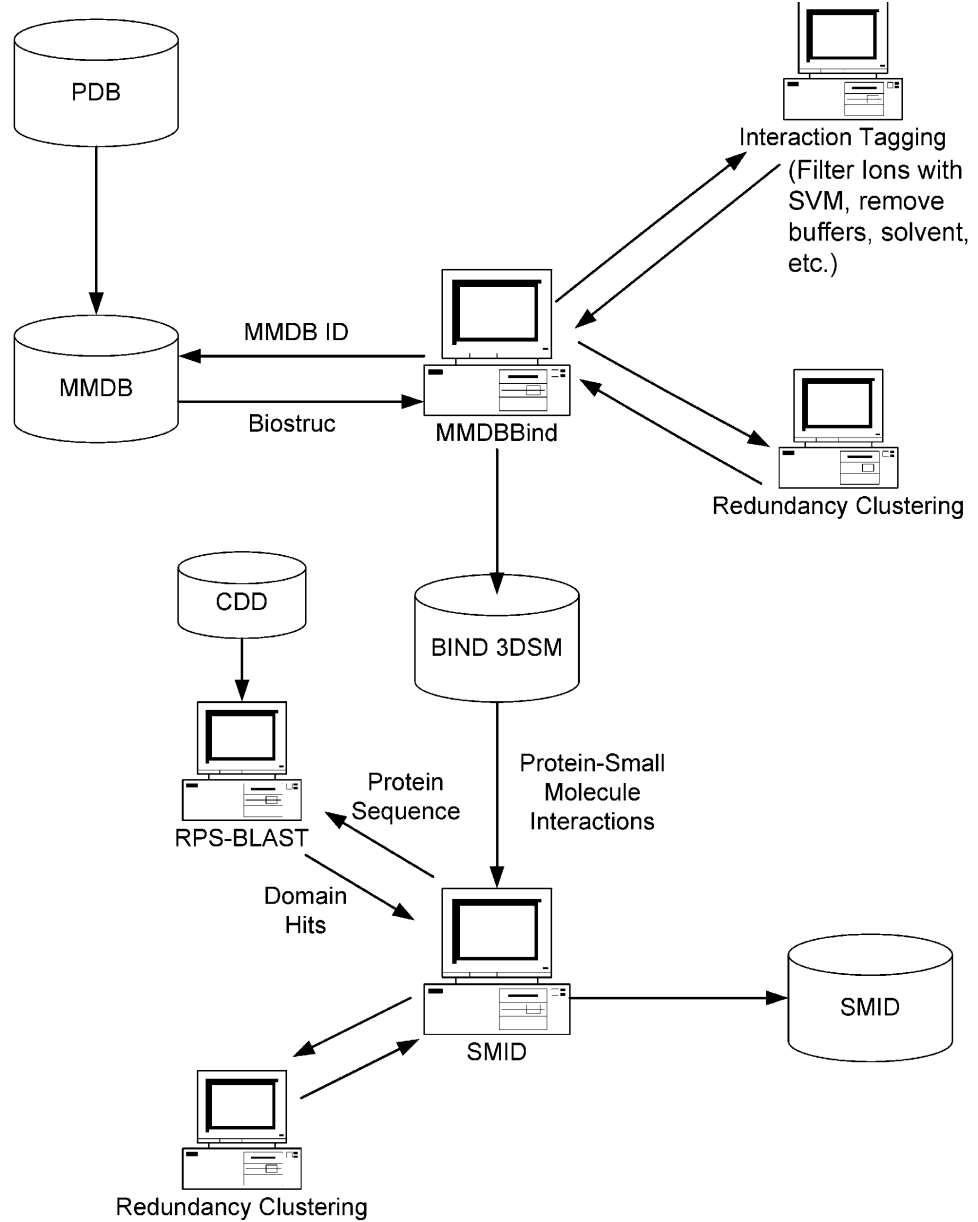
**An overview of how protein-small molecule interactions are identified from the PDB and utilized to generate SMID records**. The process of 'Interaction Tagging' involves the identification of protein-small molecule interactions that involve i) single atom contacts ii) an unknown protein sequence iii) a biologically irrelevant small molecule iv) false contacts with biologically relevant ions using a Support Vector Machine. See text for details.

An outline of the process for generating SMID records is shown in Figure 8. RPS-BLAST [26] was run on all proteins in the MMDBBIND 3DSM division with a known sequence. RPS-BLAST was performed against CDD v2.01 using a two-pass search mode and an Expect value cutoff of 0.03. All other parameters were left at their defaults. Instances of PFAM, SMART or CD domains in the query including one or more residues contacting a small molecule are selected for SMID record generation. Additionally, a minimum of two atomic contacts with the small molecule are required and a minimum of 30% of all the residues contacting the small molecule must lie within the domain in question so as to avoid interactions at the domain periphery.

Detailed information pertaining to the domain hits is extracted from the RPS-BLAST result set and stored in MySQL tables along with data on the corresponding protein and small molecule extracted from MMDBBIND. Each SMID record includes the structure from the parent MMDBBIND record, highlighting the specific domain small-molecule interaction. Interactions involving a non-biological small molecule or non-biological contacts with an ion are tagged in SMID, to enable efficient filtering of these through the interface if desired.

Redundant SMID records are clustered based on the criteria that they involve i) the same small molecule ii) the same domain and iii) a 50% or greater overlap of domain binding site residues. For example, if one interaction had a binding site on domain residues 12,13,14,15,26 and another had a binding site of 13,14,15,16, then the overlap is three residues out of five (the larger binding site is always used), or 60%. Thus these two binding sites would be considered redundant, provided the domain and small molecule are identical. Note that the overlap is always computed between the prospective redundant group member, and the parent of the redundant group (which is simply the first member of the group). Thus it is not necessarily the case that all group members mutually share 50% or greater overlap. The clustering process identified 48,886 non-redundant interactions out of a total set of 182,301.

### Ligand score

The ligand score was computed as follows:

ILS=1−log⁡10E*IS^2     (1)
 MathType@MTEF@5@5@+=feaafiart1ev1aaatCvAUfKttLearuWrP9MDH5MBPbIqV92AaeXatLxBI9gBaebbnrfifHhDYfgasaacH8akY=wiFfYdH8Gipec8Eeeu0xXdbba9frFj0=OqFfea0dXdd9vqai=hGuQ8kuc9pgc9s8qqaq=dirpe0xb9q8qiLsFr0=vr0=vr0dc8meaabaqaciaacaGaaeqabaqabeGadaaakeaacqWGjbqscqWGmbatcqWGtbWucqGH9aqpdaWcaaqaamaakaaabaGaeGymaeJaeyOeI0IagiiBaWMaei4Ba8Maei4zaC2aaSbaaSqaaiabigdaXiabicdaWaqabaGccqWGfbqrcqGGQaGkcqWGjbqsaSqabaaakeaacuWGtbWugaqcamaaCaaaleqabaGaeGOmaidaaaaakiaaxMaacaWLjaWaaeWaaeaacqaIXaqmaiaawIcacaGLPaaaaaa@4290@

where ILS is the initial ligand score, E is the RPS-BLAST E-value of the domain hit, I is the PDB identity score, and S is the relative entropy score. The latter two quantities are defined as follows.

The PDB identity score I is computed for each putative binding site by aligning the query to the PDB sequence from which the binding site was inferred, through the domain consensus. The number of exact residue matches between the query and PDB sequence for all binding site residues is computed and divided by the actual number of the PDB binding site residues to arrive at a percent identity. This gives an idea of how well the query conserves the PDB binding sites. If there is more than one example of this interaction in PDB, the score is computed for all of them, and the best (highest) one is used.

The relative entropy score was determined by computing the Shannon entropy [68] of each column of the multiple alignment for the domain family. This is then averaged across the whole alignment, and additionally over just the binding site residues (mapped from the PDB example to the domain multiple alignment). The relative entropy Ŝ is the average entropy of the binding site residues in the alignment divided by the average entropy of the full alignment. Thus a value of 1.0 indicates that the binding site residues are as well conserved as the rest of the domain sequence. A value above 1.0 means the binding site residues are less conserved than the rest of the domain, and a value below 1.0 indicates the binding site residues are more conserved on average that the rest of the domain. The latter case implies that these residues serve some important function in this domain, such as being a small molecule binding site or active site. It is important to note that the absolute value of the entropy is not considered here – the domain may or may not be well-conserved. The score only measures whether the binding site is more conserved than the rest of the sequence for this particular domain, and thus behaves as a domain-specific term with respect to the ligand. Also note that this value is independent of the query and only a function of the binding site on the domain. This term accounts for the fact that catalytic sites are under evolutionary constraints and often more highly conserved [69].

The final ligand score is computed by incorporating what we have termed the binding site occupancy. The occupancy is calculated by first grouping small molecules that appear to bind the same site on the query protein. From this listing, an occupancy value of 1 is given to the small molecule hit with the greatest number of binding site residues. The occupancies for all other small molecules in a group is determined by calculating the ratio of the number of binding site residues for a hit by the maximum number of binding site residues. For example, a particular binding site group might have a maximum number of binding site residues equal to 40 and a small molecule hit with 32 binding site residues. The occupancy for this hit would therefore be 0.8.

The final ligand score appears as a column in the summary table together with the occupancy. The score is the mean of the 'n' initial ligand scores of the hits that were clustered together to form a given entry in the summary table, multiplied by the occupancy as given in Eq. 2:

FLS=Occn*∑i=1nILSi     (2)
 MathType@MTEF@5@5@+=feaafiart1ev1aaatCvAUfKttLearuWrP9MDH5MBPbIqV92AaeXatLxBI9gBaebbnrfifHhDYfgasaacH8akY=wiFfYdH8Gipec8Eeeu0xXdbba9frFj0=OqFfea0dXdd9vqai=hGuQ8kuc9pgc9s8qqaq=dirpe0xb9q8qiLsFr0=vr0=vr0dc8meaabaqaciaacaGaaeqabaqabeGadaaakeaacqWGgbGrcqWGmbatcqWGtbWucqGH9aqpdaWcaaqaaiabd+eapjabdogaJjabdogaJbqaaiabd6gaUbaacqGGQaGkdaaeWbqaaiabdMeajjabdYeamjabdofatnaaBaaaleaacqWGPbqAaeqaaaqaaiabdMgaPjabg2da9iabigdaXaqaaiabd6gaUbqdcqGHris5aOGaaCzcaiaaxMaadaqadaqaaiabikdaYaGaayjkaiaawMcaaaaa@46D5@

Thus the occupancy factors linearly into the final computed ligand score for each binding site. This accounts for the fact that true binding pockets are often completely filled by their intended ligands. Given two small molecules with a common binding site, and all other things being equal, the one making more contacts with the protein is filling more of the binding site and so is probably a preferred ligand. Including this term in the ligand score tends to maximize the interaction surface.

### SMID-BLAST validation

In an effort to quantify the predictive power of SMID-BLAST, a validation procedure was implemented using crystal structures, MMDB ids 29251 to 32708, released after the last SMID update. A set of 2379 unique protein chains was identified for analysis using NCBI's non-redundant PDB chain file [70]. The identity tolerance level was used so that only chains identical in sequence were considered redundant. Groups involving one or more chains from structures outside the MMDB range 29251–32708 were not considered in an effort to avoid trivial interaction predictions.

The experimental and predicted interaction sets were generated by running the MMDBBIND and SMID-BLAST algorithms, respectively, on the unique protein chain set. SMID-BLAST was run using a version of the CDD compiled at the time of the last SMID update, before any of the query crystal structures had been released. Interactions involving small molecules not previously observed in SMID, or deemed to be non-biological based on the curated list used to filter MMDBBIND interactions, were excluded from further analysis. The combined small molecule and domain interaction filtering resulted in a validation dataset of 599 protein chains [see Additional file 4] involved in 860 'true' non-redundant small molecule interactions. SMID-BLAST made predictions for 581 out of 599 (97%) of the new sequences, and these make up the validation dataset (the 581 sequences comprised 793 observed interactions). Two measures were used to compare the observed and predicted interaction sets. The first measure determined the number of experimental ligands that were correctly predicted. The accuracy of the scoring method was estimated by the rank position of the exact ligand from the list of possibilities, provided that it had at least one binding site residue overlap. The second measure determined the number of correctly predicted binding site residues out of the total possible binding site residues.

## Availability and requirements

SMID is freely accessible via a PHP web interface at . The SMID data can be downloaded as tab-delimited files from our ftp server , along with a script to create and populate the MySQL tables of the SMID schema. A command line version of the SMID-BLAST tool is freely available to academic users from our ftp server . Commercial users in both cases will require a license and should contact the authors.

## Abbreviations

BIND – Biomolecular Interaction Network Database

CDD – Conserved Domain Database

PDB – Protein Data Bank

PGM – Phosphoglycerate mutase

PSSM – Position Specific Scoring Matrix

RPS-BLAST – Reverse Position Specific BLAST

SAS – Sequence Annotated by Structure

SMID – Small Molecule Interaction Database

TrpRS – Tryptophanyl tRNA synthetase

TyrRS – Tyrosyl tRNA synthetase

## Authors' contributions

Coding for the MMDBBIND, SMID and SMID-BLAST algorithms, along with the design of the SMID web interface, was carried out by KS, HF and JS. The SMID-BLAST ligand score was conceived by HF. The SMID-BLAST validation scheme was conceived and carried out by MD, KS and HF. The SMID-BLAST example queries were provided by KS, HF and CWVH. All authors read and approved the final manuscript.

## Supplementary Material

Additional File 1 Definition lines of TrpRS sequences used in Table 3 Click here for file

Additional File 2 Definition lines of TyrRS sequences used in Table 3 Click here for file

Additional File 3 Non-biological small molecule filter list used by MMDBBIND Click here for file

Additional File 4 PDB identifiers and chains for the SMID-BLAST validation test set Click here for file

## Figures and Tables

**Table 1:** SMID-BLAST hits for *Burkholderia pseudomallei *K96243 tRNA thiotransferase. For clarity, only small molecule hits with a final ligand score above the cutoff value of 50 are included. Molecule 3-letter names were obtained from PDBSum [71].

**Molecule Identifier**	**Binding Site Residues On Query**	**Final Ligand Score**
**F4S**	157,159,161,163–165,167–168,200–202,207–208,242–244,281	181.641
**SAM**	151,153,163–166,199–202,205–208,240–244, 267,269,281,288,307,309–310,338–341,350	108.434

**Table 2:** SMID-BLAST hits for *Mycobacterium tuberculosis *CDC1551 Phosphoglycerate Mutase. For clarity, only small molecule hits with a final ligand score above the cutoff value of 50 are included. Molecule 3-letter names were obtained from PDBSum [71].

**Molecule Identifier**	**Binding Site Residues on Query**	**Final Ligand Score**
**IP6**	11–12,14–15,17–18,22–24,93,101,117–118,206	897.76
**VO3**	11,18,23–25,90–91,93,101,117–118,185	804.199
**3PG**	11–15,18–19,24,63,90,183,209	799.119
**Citric acid**	11,18,22–25,63,90,93,101,117	690.171
**FDQ**	11–12,18,23,63,90,153,183–184,188	555.168
**VA3**	11–12,18,24,63,90,183–185	528.282
**benzene hexacarboxylic acid**	11,18,93,117–118,185,206	437.423
**SO1**	12,14–15,18–19,24	257.242
**B-D-Fructose-6-P**	23–24,90,101,115,119,153,184,188	134.283
**GTP**	23,90–91,101,112,115,119,124,153,184–185,188	124.075
**G3P**	101,115,119,153,184–185,188	62.337

**Table 3:** Top scoring SMID-BLAST ligand hits for TrpRS and TyrRS across a wide range of organisms. 82 TrpRS sequences and 83 TyrRS sequences were employed. The expected best ligand is shown in bold.

	Ligand to TrpRS	Hits	Ligand to TyrRS	Hits
Top scoring ligand, ignoring ATP and Mg^++^	**Tryptophanyl Adenylate**	80	**Tyrosinal Adenylate**	37
	Tyrosinal	2	SB-239629	18
			Tryptophanyl Adenylate	13
			SB-243545	9
			SB-284485	5
			D-tyrosine	1
Top scoring ligand, ignoring ATP, Mg^++ ^and binding site occupancy	**Tryptophanyl Adenylate**	36	SB-239629	50
	L-tryptophan	22	D-tyrosine	31
	D-tyrosine	10	**Tyrosinal Adenylate**	1
	SB-239629	7	Tyrosinal	1
	Tyrosinal	6		
	L-tryptophanamide	1		
